# Statin therapy in critical illness: an international survey of intensive care physicians’ opinions, attitudes and practice

**DOI:** 10.1186/1472-6904-12-13

**Published:** 2012-06-28

**Authors:** Manu Shankar-Hari, Peter S Kruger, Stefania Di Gangi, Damon C Scales, Gavin D Perkins, Danny F McAuley, Marius Terblanche

**Affiliations:** 1 Division of Asthma, Allergy and Lung Biology, King’s College London, London, UK; 2 Critical Care and Anesthesia Research Group, King’s Health Partners Academic Health Sciences Centre, London, UK; 3 Department of Critical Care Medicine, Guy's and St Thomas' NHS Foundation Trust, London, UK; 4 Princess Alexandra Hospital, Wooloongabba, Brisbane, Australia; 5 University of Queensland, Brisbane, Australia; 6 Interdepartmental Division of Critical Care, University of Toronto, Toronto, Canada; 7 Department of Critical Care Medicine, Sunnybrook Health Sciences Centre, Toronto, Canada; 8 Warwick Clinical Trials Unit, Warwick Medical, University of Warwick School, Warwick, UK; 9 Centre for Infection and Immunity, The Queen’s University of Belfast, Belfast, UK

**Keywords:** Survey, Statin, Sepsis, Critical care, Clinical trials

## Abstract

**Background:**

Pleotropic effects of statins on inflammation are hypothesised to attenuate the severity of and possibly prevent the occurrence of the host inflammatory response to pathogen and infection-related acute organ failure. We conducted an international survey of intensive care physicians in Australia, New Zealand (ANZ) and United Kingdom (UK). The aims of the survey were to assess the current prescribing practice patterns, attitudes towards prescribing statin therapy in critically ill patients and opinions on the need for an interventional trial of statin therapy in critically ill patients.

**Methods:**

Survey questions were developed through an iterative process. An expert group reviewed the resulting 26 items for face and content validity and clarity. The questions were further refined following pilot testing by ICU physicians from Australia, Canada and the UK. We used the online Smart Survey^TM^ software to administer the survey.

**Results:**

Of 239 respondents (62 from ANZ and 177 from UK) 58% worked in teaching hospitals; most (78.2%) practised in ‘closed’ units with a mixed medical and surgical case mix (71.0%). The most frequently prescribed statins were simvastatin (77.6%) in the UK and atorvastatin (66.1%) in ANZ. The main reasons cited to explain the choice of statin were preadmission prescription and pharmacy availability. Most respondents reported never starting statins to prevent (65.3%) or treat (89.1%) organ dysfunction. Only a minority (10%) disagreed with a statement that the risks of major side effects of statins when prescribed in critically ill patients were low. The majority (84.5%) of respondents strongly agreed that a clinical trial of statins for prevention is needed. More than half (56.5%) favoured rates of organ failure as the primary outcome for such a trial, while a minority (40.6%) favoured mortality.

**Conclusions:**

Despite differences in type of statins prescribed, critical care physicians in the UK and ANZ reported similar prescription practices. Respondents from both communities agreed that a trial is needed to test whether statins can prevent the onset of new organ failure in patients with sepsis.

## Background

Host immune and inflammatory response to infection manifests as sepsis syndromes [1]. Sepsis is common and its incidence appears to be increasing[2,3], accounting for 27% of United Kingdom (UK) and 12% of Australia-New Zealand (ANZ) critical care admissions [4]. Worryingly the reported case fatality rate from sepsis syndromes remains high (25%-50%) [5]. The estimated annual cost of treating sepsis in the United States was $16.7 billion [5], whilst the estimated cost of managing a patient with sepsis in the intensive care unit has been reported to vary between $19,000 to $28,000 [6]. Furthermore, interventions such as resuscitation of patient with sepsis further adds to the management cost [7].

The biology of sepsis syndrome is characterised by unregulated systemic inflammation and immune dysfunction involving multiple pathways [8]. Numerous studies have tested the efficacy and effectiveness of immune-modulating agents; so far all have failed to show benefit. One reason may be that modulating a single immune target, embedded in a complex system with multiple redundancy, is insufficient to effect a subsequent improvement in morbidity or mortality[9]. Testing agents with modulating effects at multiple points may therefore be a more successful strategy [10-14] to prevent the onset of acute organ failure.

Statins are 3-hydroxy-3-methylglutaryl coenzyme A (HMG-CoA) reductase inhibitors with an established role in primary and secondary prevention of cardiovascular events by lowering low-density lipoprotein cholesterol [15]. Recent mechanistic reviews show statins influence inflammatory pathways at multiple levels through effects on cellular signalling pathways independent of lipid lowering ability [16,17]. In addition to the potential role in treating critically ill patients with established organ dysfunction like sepsis syndromes [14] and acute lung injury [13], statins have also been proposed as a potential intervention to prevent new organ dysfunction in sepsis [18].

HMGCoA reductase catalyses the rate limiting step in the production of cholesterol by inhibiting the conversion of HMG CoA to mevalonate. As a consequence of this action, the intermediates of the mevalonate pathway are also reduced. Under normal circumstances the mevalonate pathway leads to the formation of isoprenoids which regulate the lipid modification of proteins necessary for interaction with cellular membranes which drive inflammatory responses. Inhibition of isoprenoid formation by statins therefore, has significant anti-inflammatory effects. These have been demonstrated in vitro and in vivo as well as in a human model of pulmonary inflammation induced by inhaled endotoxin [19]. Enzymes of the mevalonate pathways are also key for gram positive bacterial infection pathogenesis and are considered potentially modifiable with statin therapy [20]. Statins could potentially improve bacterial clearance by neutrophils and macrophages via novel extracellular traps linked to sterol pathway inhibition [21]. Antibacterial activity of compounds is often reported in terms of minimum inhibitory concentrations (MIC). However, the MICs required for antibacterial activity of statins invitro is much higher (approximately 1000 times) than what is achieved during conventional dosing for lipid homeostasis [22,23]. Therefore, the current thinking is that the antibacterial bacterial effects of statins are minimal at conventional doses and they are likely to be concentration independent, complex and as yet not fully clarified [22,24].

The literature on statin and sepsis is predominantly comprised of observational studies with inconsistent results and characterized by methodological limitations [25-27]. A recently published systematic review and meta analysis identified a potential publication bias in the existing literature on statin therapy and infections, and suggested that such publication bias might explain the inconsistent results from previous research [28]. These findings serve to highlight the need for well-designed interventional trials of statins to clarify their potential benefits in modulating the response to infections [29].

There is paucity of published evidence on the current critical care practices of statin prescribing. We conducted an international survey of critical care units in the United Kingdom (UK) and Australia & New Zealand (ANZ) to study current practice patterns of statin prescription in the critical care setting and to obtain background information to help inform the design of an interventional trial testing the hypothesis that statin therapy can prevent new acute organ failure.

## Methods

### Study participants

We targeted critical care physicians in UK and Australia identified using mailing lists maintained by The Intensive Care Society in the UK and The Australian and New Zealand Intensive Care Society Clinical Trials Group.

### Survey development

Study investigators generated potential items for the survey by reviewing the literature on sepsis and statins and by seeking expert opinions [16,30]. Item reduction was achieved using an iterative process involving all 7 study investigators, 5 other critical care physicians and 2 external appraisers. The survey questionnaire was pilot tested using a group of senior critical care physicians and researchers from Canada, UK and ANZ (n = 15) to further refine and finalise question stems and response formats, and to assess its content, face validity and clarity [31]. The final survey questionnaire consisted of 26 items arranged into 3 domains. The online Smart Survey^TM^ software was used to format the questionnaire prior to administration [32]. The research and ethics committee at Guy’s and St Thomas’ Hospital NHS Foundation Trust considered the study to pose minimal risk and waived the need for formal ethical review.

### Survey administration

Potential respondents were initially contacted by email. The email included a cover letter explaining the purpose of the survey and a link to the web-based Smart survey tool. A follow up reminder was sent 4 weeks after the initial mailing. No incentives were provided for responding to the survey.

### Survey overview

The 3 survey domains were aimed at (1) understanding the respondent demographics and the intensive care unit characteristics including management policy, (2) determining the current practice patterns of statin therapy in critical illness, and (3) gathering opinions on key design issues for a proposed interventional trial of statin therapy in early critical illness.

The intensive care units management policy was defined using the following terms: a) ‘open’ refers to the policy where the referring physician remains directly responsible for majority of day to day patient care; b) ‘closed’ refers to the policy where the referring physicians hand over care to critical care physicians who are responsible for majority of day to day patient care until critical care discharge; and c) hybrid refers a combination of open and closed.

The current practice patterns of statin therapy were evaluated using scenarios in two separate contexts: 1) *initiating* new statin prescriptions (i.e. patients who are statin-naive), and 2) continuing pre admission prescriptions following admission to critical care. The likelihood with which new statin prescriptions are initiated following critical care admission in patients’ NOT previously receiving statins was evaluated using the following 3 clinical scenarios: (1) a new cardiac indication during critical care admission; (2) preventing organ dysfunction in sepsis; and (3) treating organ dysfunction in severe sepsis. The frequency with which pre admission statin prescriptions are continued following critical care admissions for patients who were previously receiving statins was evaluated using the following 4 clinical scenarios: (1) outpatient indication like hyperlipidaemia without a new acute cardiac indication; (2) a new acute cardiac indication during critical care admission (e.g. acute coronary syndrome); (3) for preventing organ dysfunction in sepsis; and (4) for treating organ dysfunction in severe sepsis.

### Statistical analysis

We report responses to survey questions overall and by country (UK and ANZ). Institutional and ICU characteristics are described using proportions. Likert format response items were used to evaluate the reported statin prescribing practice and opinions on trial design. Reported practice and opinions evaluated using 5-point Likert scales are graphically represented by grouping responses at the tails (i.e. 1 and 2 grouped, 4 and 5 grouped). The grouping 1/2 refers to ‘frequently/always’ and grouping 4/5 refers to ‘infrequently/never’; the remaining value 3 refers to ‘sometimes’ in the ‘Likert Scale’ choices used to seek opinions. The Chi square test (and Fisher exact test when number of observations in any category was less than 5) was used to evaluate the differences between responses from UK and ANZ physicians.

## Results

### Survey respondents

We received a total of 239 responses (177 from UK and 62 from ANZ). More respondents (overall 58.2%; n = 139; UK: n = 86, ANZ: n = 53) practised at university or teaching hospitals than at district general or community hospitals whilst 3 respondents did not specify the hospital type (overall 40.6%; n = 97; UK: n = 88, ANZ: n = 9). The ICUs in these hospitals had a median (IQR) 16 (13) [UK = 15 (11) and ANZ 18 (12)] critical care beds admitting a median (IQR) 900 (870) [UK = 750 (700) and ANZ = 1225(1100)] patients annually. The management policy for treating patients was described as closed by 78.2% (overall n = 187; UK: n = 133, ANZ: n = 54) and as hybrid by 21% (overall n = 50; UK: n =42, ANZ: n = 8) of respondents. The case-mix of patients was described as ‘predominantly medical’ by 12.2%’, and ‘predominantly surgical’ by 11% and as ‘mixed medical and surgical’ by 71.3% (overall n = 169; UK: n = 124 & ANZ: n = 45) of the respondents,

### Current statin prescription practices in critical care setting

The most frequently prescribed statins were simvastatin (overall 65.2%; UK = 77.6% *vs.* ANZ = 28.8%; p < 0.001) and atorvastatin (overall 28.3%; UK = 15.5% *vs.* ANZ = 66.1%; p < 0.001). The most common reason cited for choosing a particular statin from both the UK and ANZ physicians was prior use (overall 56.1%; UK 54.9% *vs.* ANZ 60.0%; p = 0.423). Other reported reasons included availability from hospital pharmacy (23.6%), drug policy (6.3%) and cost (9.9%). There was no statistically significant difference when comparing responses from UK and ANZ (p = 0.682). Overall, nearly half of respondents agreed or strongly agreed that the risks of major side effects of statins (i.e. increase in hepatic transaminases, elevated creatine kinase, rhabdomyolysis) are low when prescribed in critically ill patients (UK 48.6% *vs.* ANZ 50.0%). There was no statistically significant difference between UK and ANZ physicians’ opinions on statin safety (p = 0.291).

### Initiating new statin prescription following critical care admission

**Figure 1:**
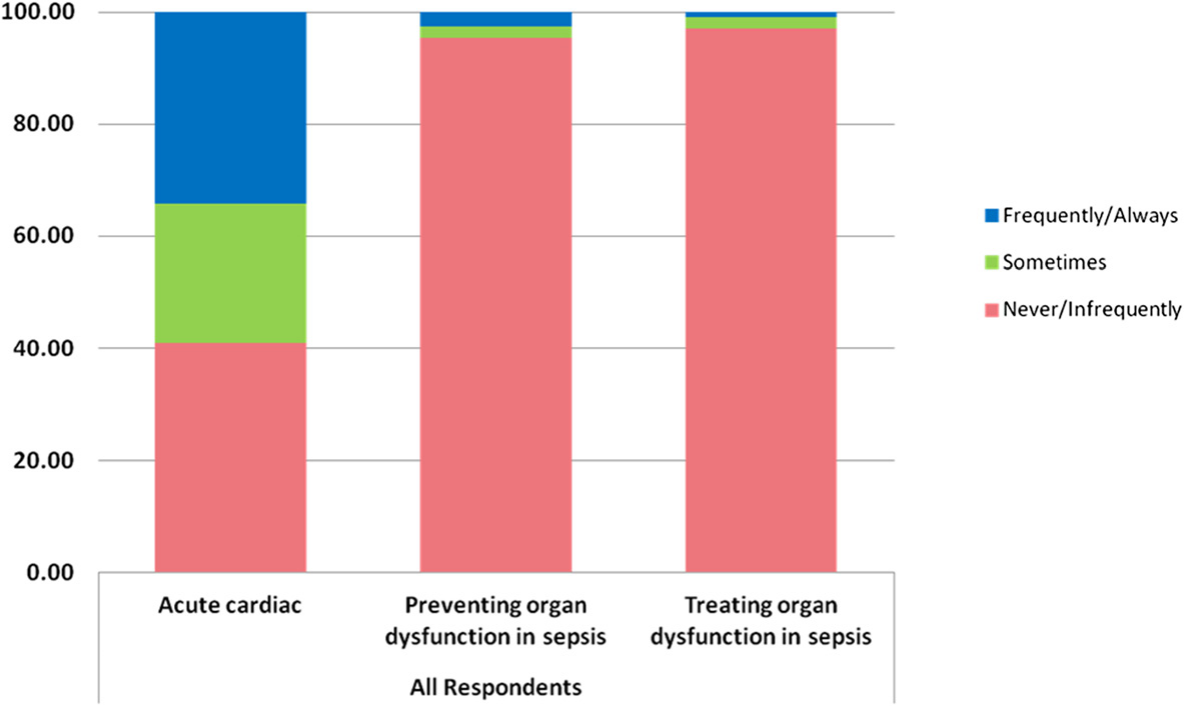
New statin prescription practice following critical care admission.

Overall 34.3% of all respondents stated they would start a new statin prescription for a new cardiac indication during a patient’s ICU admission (35.6% UK *vs.* 30.7% ANZ; p = 0.26). Respondents reported rarely starting a new statin prescriptions to prevent organ dysfunction in sepsis or to treat organ dysfunction in severe sepsis (overall 2.5%; 1.1% UK *vs.* 6.4% ANZ; p = 0.03) and overall 0.8%; 0.6% UK *vs.* 1.6% ANZ; p = 0.75 respectively) [Figure 1].

### Continuing pre admission statin prescriptions following critical care admission

**Figure 2:**
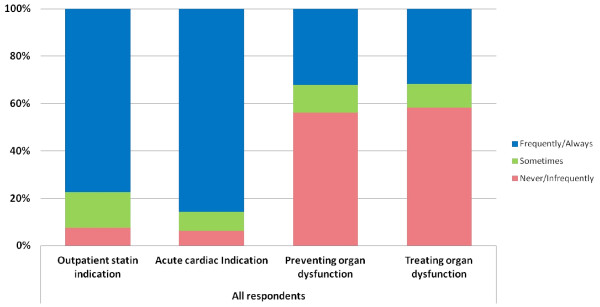
Continuing statin prescription practice following critical care admission.

Most respondents stated they would continue preadmission statin prescription for outpatient indications even if there were no new cardiac indications in the current ICU admission(overall 77.4%; 83.6% UK *vs.* 59.7% ANZ; p < 0.001). Similarly, most respondents reported that they would continue pre admission statin prescriptions if there was a new cardiac indication in the current admission (overall 85.8%; 87.0% UK *vs.* 82.3%ANZ; p = 0.18). However, fewer respondents would continue pre admission statin prescriptions for preventing organ dysfunction in sepsis or for treating organ dysfunction in severe sepsis (overall 32.2%; 31.6% UK *vs.* 33.9% ANZ; p = 0.87 and overall 31.8%; 32.8% UK *vs.* 29.3% ANZ; p = 0.72 respectively) [Figure 2].

### Statin interventional trial

#### Hypothesis and trial participation

Overall, most respondents stated that they ‘neither agree or disagree’ that statin therapy can prevent the onset of acute organ failure in critically ill patients (overall 81.2%; UK 80.8 *vs.* 82.3% ANZ; p = 0.11). Importantly, most respondents either agreed or strongly agreed that there is a need for a randomised clinical trial to test the hypothesis that statin therapy prevents acute organ failure in critical illness (overall 84.5%; UK 84.2% *vs.* ANZ 85.5%; p = 0.56). The majority of UK physicians (75.1%) indicated a willingness to participate in a therapeutic trial evaluating the effect of statins for preventing organ failure in critical illness. Although in contrast no ANZ physicians were willing at this stage to participate in the proposed study, the vast majority were undecided (overall 36.0%; 17.5% UK *vs.* 88.7% ANZ; p < 0.001). Overall, few were unwilling to participate in the proposed trial (8.4%; 7.3% UK *vs.* 11.3% ANZ).

#### Type of statin therapy and safety triggers

The preferred statins for a future interventional trial were simvastatin and atorvastatin (overall 31.9% and 29.8% respectively). Comparing the responses from physicians in the UK and ANZ, there were significant differences in the choice of statins for the proposed trial (simvastatin - 38.4% UK *vs.* 13.1% ANZ; p < 0.001 and atorvastatin - 21.5% UK *vs.* 54.1% ANZ; p < 0.001). Almost one-third (32.4%) of respondents did not report preference to a particular statin.

We ascertained respondents’ views regarding the biochemical cut offs (i.e. an increase above the upper limit of normal in hepatic transaminases, creatine kinase and/or bilirubin) below which they considered it safe to enrol patients in the proposed trial. Comparing the responses from physicians in the UK and ANZ, there were statistically significant differences in the choice of cut offs for trial enrolment with higher biochemical cut-offs tolerated by physicians from ANZ (Table 1). Using three-times upper limit of normal as an example to demonstrate the heterogeneity in the choice of cut offs we observed for: 1) transaminase levels - overall 80.7%; UK = 83.5% *vs.* ANZ = 74.2%; p = 0.007; 2) serum creatine kinase levels – overall 79.2%; UK = 82.8% *vs.* ANZ = 71.0%; p < 0.001); and 3) serum total bilirubin levels – overall 82.6%; UK = 86.2% *vs.* ANZ = 74.2%; p = 0.003).

**Table 1:** Cut offs for biochemical tests below which the respondents would enrol patients into the proposed trial

**THRESHOLD**	**OVERALL (%)**			**UK (%)**			**ANZ (%)**		
	**ALT/AST**	**CK**	**BILIRUBIN**	**ALT/AST**	**CK**	**BILIRUBIN**	**ALT/AST**	**CK**	**BILIRUBIN**
uln	27.6	34.7	33.9	31.6	41.8	38.4	16.1	14.5	21.0
3 uln	42.3	33.9	37.7	36.7	26.0	32.2	58.1	56.5	53.2
5 uln	13.8	13.4	12.1	11.3	10.7	8.5	21.0	21.0	22.6
10 uln	2.5	2.9	1.7	2.3	2.8	1.7	3.2	3.2	1.6
>10 uln	0.4	1.7	1.3	0	0.6	1.1	1.6	4.8	1.6
missing/no response	13.4	13.4	13.4	18.1	18.1	18.1	0	0	0
total	100	100	100	100	100	100	100	100	100

Shows the biochemical cut offs for hepatic transaminases (ALT/AST), creatine kinase (CK) and total bilirubin (bilirubin) below which the respondents would consider enrolling patients in the proposed statin interventional trial. Data are presented as proportions. ULN = upper limit of normal. p = 0.007 for ALT/AST; p < 0.001 for CK; p = 0.003 for Bilirubin (p-values when comparing the overall frequency distribution of the responses for ANZ and UK).

#### Trial end points

The majority of respondents (56.5%) preferred rates of new organ failure as the most appropriate primary outcome of choice for power calculations and there were no statistically significant differences when comparing responses between UK and ANZ (60.7% UK *vs.* 46.8% ANZ; p = 0.06). However, 40.6% of respondents felt that mortality was the most appropriate primary outcome of choice for power calculations and there were statistically significant differences when comparing responses between UK and ANZ (35.2% UK *vs.* 53.2% ANZ; p = 0.02). There were statistically significant differences in the choice of follow up duration between the UK and ANZ (e.g. for 28 day follow up – overall 33.3%; UK = 37.9% *vs.* ANZ = 22.6%; p = 0.032) [Table 2].

**Table 2:** Preferred follow up duration for the proposed statin interventional trial

**follow up duration**	**overall - %**	**uk - %**	**anz - %**	p*
1-7 days	1.9	1.4	3.2	0.585
8-14 days	3.4	4.1	1.6	0.677
15-21 days	2.4	2.8	1.6	1.000
21-28 days	33.3	37.9	22.6	0.032
3 months	29.0	22.8	43.6	0.003
6 months	26.1	28.3	21.0	0.304
other	3.9	2.8	6.5	0.244
total	100	100	100	

Shows respondents’ preferred follow up duration for the proposed statin interventional trial. Data are presented as proportions. ^*^ p value for comparison of United Kingdom versus Australia and p = 0.024 when comparing the overall frequency distribution of the responses for ANZ and UK.

## Discussion

### Key findings

We surveyed critical care physicians from a broad sample of critical care units in the UK and ANZ to obtain a cross section of statin prescribing practices in critically ill patients. The principal differences in reported statin prescription practices related to decisions to continue prior prescriptions, whether to initiate a new prescription, and about the degree of biochemical abnormalities physicians would tolerate when prescribing a statin. A significantly higher proportion of UK critical care physicians continued statins for outpatient indications in ICU, whereas more ANZ critical care physicians were likely to initiate new statin prescriptions to prevent organ dysfunction in sepsis. Interestingly, only a third of respondents would initiate a statin prescription for an acute cardiac indication in critically ill patients, whilst nearly a third of respondents would continue a pre existing statin prescription for preventing or treating organ dysfunction secondary to sepsis syndromes.

Critical care physicians in both ANZ and UK agreed that a randomized controlled trial is needed to test the efficacy of statins for preventing organ dysfunction in early critical illness. However, while the majority of UK physicians would be willing to participate in such a trial, the majority of ANZ physicians were undecided based on the description of the trial that was provided. We can only speculate on the reasons for these differences, but one possibility is that ANZ physicians are more accustomed to focusing on large-scale pragmatic trials that aim to improve survival [33-35]. Indeed, more ANZ survey respondents wanted the interventional trial to be powered for mortality as the primary outcome, whereas more UK respondents preferred organ failure. The preferred statin for such an interventional trial was simvastatin in the UK and atorvastatin in ANZ, possible reflecting different prescribing practices between the two jurisdictions. Higher biomarker thresholds for stopping and initiating statin therapy were also reported by physicians in ANZ compared to those in the UK. These important differences must be considered when designing interventional trials of statin therapy in critical illness.

### Strengths and weaknesses

The survey objectives were to evaluate opinions on an active area of critical care research in critical illness, i.e. the potential role of statins to attenuate or prevent organ dysfunction through the modulation of inflammation. This survey provides important information on the ICU community’s views on this topic. The methodological rigour in the design of this survey, including careful sensibility testing and pilot testing, should reduce the potential for bias, a common problem in survey research [36]. We also targeted physicians in two large international regions to ensure we had broad representation from the critical care community and to improve generalisability.

Our study has several limitations that are common to most surveys. First, as with most self-administered surveys, our response rate was incomplete. Although, we used the official mailing lists there are no data on completeness and accuracy of mailing lists. Furthermore, these mailing lists include non physician health care staff and trainee doctors who were not our target population. Although these groups were actively excluded the total number at any one time is variable. However, the survey responses represent replies from 74.6% of UK adult general critical units and 37.1% of ANZ critical care units. Second, our results reflect the reported practice of respondents, and may not reflect actual practice. Third, we are unable to rule out response bias, and some of the non-responding physicians may have provided different responses to survey questions.

## Conclusions

Significant variability exists in the reported statin prescription practices in decisions to continue prior prescriptions, to initiate a new prescription, and in the degree of biochemical abnormalities physicians would tolerate when prescribing a statin. Our results, reflecting the opinions of critical care physicians across UK and ANZ, confirm that equipoise exists around the utility of statins to prevent and/or treat organ dysfunction in the critically ill patient population. Critical care physicians from both regions agreed that a clinical trial is needed to clarify the role of statins in this patient population.

## Abbreviations

UK, United Kingdom; ANZ, Australia and New Zealand; ICU, Intensive care unit; RCT, Randomised controlled trial.

## Competing interests

The authors declare that they have no competing interests.

## Financial support

DCS is supported by a New Investigator Award from the Canadian Institutes for Health Research.

MT acknowledges the support of the UK NIHR Biomedical Research Centre scheme.

## Authors' contributions

All authors contributed equally to the conception and design of the study, interpretation of data, and critical revision of the manuscript. SG and MS-H developed the analysis plan and participated in the analysis. All authors read and approved the final manuscript.

## Pre-publication history

The pre-publication history for this paper can be accessed here:

http://www.biomedcentral.com/1472-6904/12/13/prepub
